# Eating together as a social network intervention for people with mild intellectual disabilities: a theory-based evaluation

**DOI:** 10.1080/17482631.2018.1516089

**Published:** 2018-09-11

**Authors:** Kasper Kruithof, Jeanine Suurmond, Janneke Harting

**Affiliations:** Department of Public Health, Amsterdam UMC, University of Amsterdam, Amsterdam, The Netherlands

**Keywords:** Mild intellectual disabilities, theory-based evaluation, participant observation, social work, social network intervention, communal dining

## Abstract

**Purpose:** People with mild intellectual disabilities (MID) generally live independently among the wider community. This can result in social exclusion and feelings of loneliness. Therefore, social work organizations aim to socially include people with MID through organizing activities in neighbourhoods that should lead to enlarged networks and increased societal participation. The “Communal Table” is such a, group-orientated, intervention that organizes monthly dinners in Amsterdam, the Netherlands. Because little is known about the effectiveness of interventions aiming to bring about social inclusion for people with MID we explored which types of participants were reached and whether and how the intervention brought about the intended outcomes.

**Methods:** We performed a theory-based evaluation, using participatory observations and qualitative interviews (n = 19). The Communal Table attracted a diverse and loyal group of participants.

**Results:** We distinguished four types of participants—lonely participants, activist participants, satisfied participants and calculating participants—whose pre-existing networks played a significant role in their individual needs for support and the outcomes of the intervention. Outcomes reported included experiences of conviviality and warmth, temporary relief of underlying problems and an overall positive opinion about the intervention, but network enlargement or increased societal participation were not reported.

**Conclusions:** Our findings suggest that social network interventions for people with MID should be tailored to participants’ pre-existing networks and related individual needs to be successful.

## Introduction

### Background

Self-reliance and societal participation are generally difficult to obtain for people with mild intellectual disabilities [MID] (Kwekkeboom, De Boer, Van Campen, & Dorrestein, 2006; Wilken, Medar, Bugarszki, & Leenders, 2014). In addition, they often lack social networks to support them in these respects (Robertson et al., 2001; Verdonschot, de Witte, Reichrath, Buntinx, & Curfs, 2009; Verplanke & Duyvendak, 2009). Since self-reliance, societal participation and taking responsibility for one’s own life are the main pillars of contemporary Western welfare societies (Newman & Tonkens, 2011), and deinstitutionalization is a trend in these societies (Braddock, Emerson, Felce, & Stancliffe, 2001; Coucouvanis, Lakin, Prouty, & Webster, 2006; Grunewald, 2003; Mansell, 2006; McConkey, Abbott, Walsh, Linehan, & Emerson, 2007; Stancliffe, Lakin, & Prouty, 2005), social work organizations have started to support people with MID in building social networks for support, so they can achieve societal participation and become more self-reliant (Newman & Tonkens, 2011). Deinstitutionalization and extramuralization can be viewed as empowering for vulnerable groups, but can result in social exclusion and feelings of loneliness as well (Verplanke & Duyvendak, 2009). Larger social networks and increased societal participation should lead to social inclusion, a higher quality of life and increased mental well-being for people with MID (Berkman, 2009; Duvdevany & Arar, 2004; Schalock, 2004).

Little is known about the effect of social work interventions that aim to bring about network enlargement and increased societal participation for people with MID. There have been studies on individual trajectories for people with MID (Howarth, Morris, Newlin, Webber, & Newlin, 2016; van Asselt-Goverts, Embregts, & Hendriks, 2016), which found that programmes providing instrumental support, such as social skills training and changes in activity patterns, are successful in enlarging the networks of the socially excluded. By contrast, group-oriented interventions are hardly represented in the scientific literature (Martina & Stevens, 2006). The scientific knowledge gap contrasts with the general literature on social work interventions, which stresses the need of focusing on groups rather than on individual characteristics of people (Loeffler et al., 2004; Lynn, 2006), given the contradiction of achieving social inclusion for the excluded by focusing on the individual (Lomas, 1998). In this study, we aimed to bridge the knowledge gap regarding group-oriented interventions intended to include the socially excluded in general, and more specifically regarding social work interventions aimed at enlarging networks and increasing societal participation among people with MID.

### Intervention

The social work intervention called the Communal Table aims at socially including people with MID in the neighbourhood by enlarging their social networks and increasing their societal participation. The intervention consists of monthly three-course dinners that are organized in different districts in Amsterdam, the Netherlands. Each district has its own Communal Table. Each Communal Table generally includes 20–30 participants, a professional social worker and about three volunteers. In each district, the intervention is organized at various community centres. Each time, a space is set up with tables laid especially for the Communal Table and its participants. Dinner starts at about 5 pm and ends at about 7 pm. The food is prepared and served by the staff of the community centre. The social work agency that offers the Communal Table pays for the food, including coffee and tea. Participants pay one euro to the agency for their three-course dinner. This price was chosen so that the Communal Table remains inclusive, while the participants feel they do not get the dinner for free. The Communal Table is offered the whole year around and everyone with MID and a desire to eat along is welcomed. The social work agency that provides the intervention sends out invitations by post every month to all eligible participants. For this purpose, the agency keeps a list of people with MID who have said to be interested in participating in the Communal Table. In addition, the social workers phone potential new participants from the list to make the threshold for participating in the Communal Table as low as possible.

**Table 1.** Characteristics of the respondents.

Name (fictitious)	Age	Communal Table A/B/C	Employment status	Type of participant
Carolina (f)	55	A	Unemployed	Lonely
Marsha (f)	62	A	Part-time work	Lonely
Joseph (m)	70	A	Retired	Lonely
Aysha (f)	23	A	Unemployed	Lonely
Jan (m)	46	A	Employed	Activist
Sophie (f)	43	A	Employed	Activist
Simon (m)	69	A	Retired	Satisfied
Gerard (m)	52	A	Part-time work	Satisfied
Jacob (m)	53	B	Employed	Lonely
Andre (m)	63	B	Employed	Lonely
Karima (f)	42	B	Unemployed	Lonely
Toby (m)	52	B	Part-time work	Activist
Tim (m)	29	B	Part-time work	Activist
Therese (f)	39	B	Part-time work	Satisfied
Benny (m)	36	B	Unemployed	Satisfied
Tom (m)	42	C	Employed	Lonely
Hank (m)	47	C	Employed	Satisfied
Nick (m)	36	C	Employed	Satisfied
Britney (f)	41	C	Part-time work	Satisfied

### Programme theory

To enable us to evaluate the Communal Table, we drew up a programme theory for the intervention. A programme theory is a plausible model of how an intervention is expected to bring about its intended effects (Birckmayer & Weiss, 2000). For the Communal Table, we based the programme theory on a practical description of the intervention as well as on insights from social epidemiology (Berkman, Kawachi, & Glymour, 2014) and social science theories (Granovetter, 1973; Putnam, 1995)

**Figure 1.**
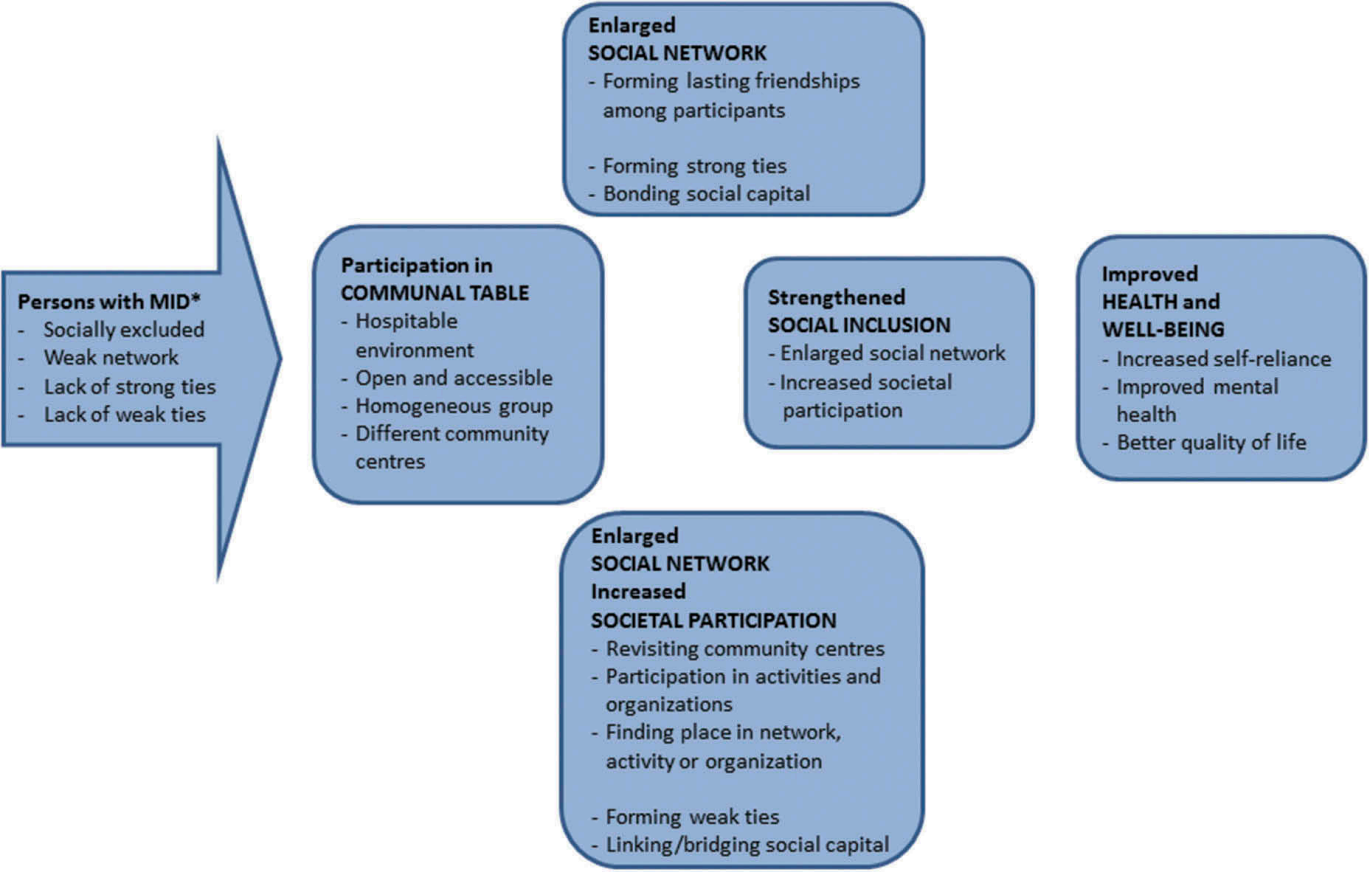
Programme theory for the social work intervention “Communal Table”. * MID = mild intellectual dissabilities. Based on Granovetter, 1973; Putnam, 1995; Berkman et al., 2014.

The Communal Table [see Figure 1] offers a hospitable environment that is easily accessible for a homogenous group of socially excluded people. In this setting, explicit and implicit mechanisms are intended to contribute to improved health and well-being (Berkman et al., 2014). Such ultimate advantages of the Communal Table may be brought about through two different mechanisms. First, by getting people together on a regular basis in a homogenous group in an open, safe and hospitable atmosphere, in which the participants will form lasting friendships among themselves. Such friendships can cause the initially weak social networks of people with MID to be enlarged with strong ties (Granovetter, 1973). Such strong ties reflect the development of trust and reciprocity, i.e., the creation of bonding social capital (Putnam, 1995). Second, participants are encouraged to take part in activities offered by a variety of organizations, by visiting different community centres with the Communal Table. In doing so, they also become socially embedded in broader networks of weak ties. Transcending the boundaries of their own social group, i.e., building bridging social capital, is expected to contribute to personal growth and problem-solving capacity (Granovetter, 1973; Putnam, 1995). People with MID may also find a permanent position in these activities in which they participate, e.g., by performing voluntary work. If participants become a functionally integrated part of an organization, this means they also build up linking social capital (Putnam, 1995).

### Aim and research questions

We explored the expected mechanisms and outcomes by focusing on the experiences and perspectives of people with MID regarding the Communal Table with the following questions in mind:

*What kind of participants did the Communal Table reach and how did the participants experience and perceive the intervention? Did the Communal Table bring about network enlargement and societal participation for its participants and, if so, did this lead to experienced social inclusion and a self-reported increase in self-reliance, quality of life, and mental well-being?*

## Method

### Design

We conducted a theory-based evaluation of the Communal Table in which we used participatory observations (Bryman, 2015). The Communal Table was visited by KK as a participant observer in the role of a volunteer. We opted for this participatory approach because we expected this to lead to greater mutual trust and openness between him and the participants (Bernard, 2011; De Munck & Sobo, 1998). In turn, this was expected to lead to a more thorough understanding of the participants’ situation, and therefore more targeted data collection, and thus to a better understanding of the intervention (De Munck & Sobo, 1998). During his presence, KK took field notes based on observations and informal talks. In addition, he invited the participants for a formal interview. This methodological triangulation was expected to result in more robust and richer data (Bryman, 2015), which is especially valuable in research with vulnerable groups that often have difficulties expressing their thoughts and emotions in a coherent way (Russell, 1999).

### Data collection

Between April 2016 and December 2016, three different Communal Tables [called A, B and C below] were visited a total of 16 times. At the start of our field work, Communal Table A had existed for 18 months. Communal Table B had been founded about a year before the field work started. Communal Table C was relatively new and had existed for half a year before the field work started.

### Field notes

Field notes of observations and informal talks, by KK in his role as participant-observer (Gold, 1958), were entered as notes (Bryman, 2015) in a smartphone, in a discreet manner. This was not done to hide the fact that there was research going on, but in order not to make the people aware that they were being observed, as that could lead to them acting differently or cautiously. Focal points were: interaction between participants, interaction between participants and professionals or volunteers, times of arrival and departure, and general noteworthy occurrences or events. The informal talks with participants about the Communal Table and their personal situation were intended to result in a broader understanding of the various participants. We thereby aimed for a less biased view of our research population, as this approach enabled us to include, to some degree, also the feelings and situations of people who did not participate in a formal interview. Moreover, the informal talks and observations were used in an inductive manner, in that the themes that arose from the observations were used in the interviews. This resulted, for example in discussing the arrival and departure times and the social contacts with others, or the lack thereof, in the interviews.

### Interviews

Interviews were held between October 2016 and December 2016. Nineteen participants (see Table 1), were interviewed in a semi-structured way (Bryman, 2015). The main themes addressed in interviews were general well-being, network size, societal participation, reasons to participate in the intervention, experiences with the intervention, and hopes/dreams and ambitions. The respondents were offered a certain freedom to decide the direction of the interview. If they went off-topic, this was not prevented, in order to allow the respondents to speak in their own words about their own themes; i.e., to *Verstehen* the respondents (Weber, 1947). This approach was intended to prevent that the interviewer directed the interviews too much, something that is important when interviewing vulnerable groups (Russell, 1999). The interviews took place at the home of the respondent or in a public place, like a bar or café, chosen by the respondent. All but two of the interviews were one-to-one. One respondent felt more comfortable with a friend on her side and another wanted a neighbourhood worker to be present. Interviews took between 30 and 150 minutes, with a mean of one hour. The wide range of interview durations had to do with differences in the respondents’ mental well-being. These different personal situations meant that not all topics were relevant for every respondent. For example, some respondents did not have any particular opinion about the Communal Table. They were satisfied with their lives and visited the Communal Table with no specific goal in mind. Other respondents felt lonely and wanted to talk about their experienced problems.

Participants were invited for an interview by KK during a central moment before or after dinner. Since KK knew the respondents well and “hung out” with them frequently (Bernard, 2011), no reward was offered for the interviews, with the exception of Communal Table C, which was not visited as often by KK. We hoped, by offering a reward in this case, to get to speak to a group that initially may not have been inclined to participate in an interview. Irrespective of whether they were offered a reward or not, half of the participants of each of the Communal Tables agreed to take part in an interview. Data saturation was reached after about two-thirds of the interviews.

### Medical ethics approval and informed consent

According to the Dutch Medical Research Involving Human Subjects Act, this study did not require approval by a medical research ethics committee in the Netherlands. We followed the ethical principles for medical research involving human subjects as laid down in the Declaration of Helsinki and adopted by the World Medical Association (Association, 2000). KK’s role as researcher was discussed at each Communal Table. Each participant was sufficiently informed of the aims and methods of the study and *a priori* oral consent was obtained from the participants and also audio taped. Codes were used to designate the participants to guarantee their anonymity.

### Data analysis

The interviews were transcribed literally and analysed thematically (Miles, Huberman, & Saldana, 2014; Ritchie & Spencer, 2002). Field notes were assigned to the thematic codes used for the interviews. The thematic codes were distilled from the literature, the theoretical mechanisms of the intervention and the expected outcome and mediating concepts of the intervention. This theory-led form of coding (Bryman, 2015) resulted in the following main themes: network size and network type, mental well-being, motivation to participate, inclusion and exclusion in the context of the intervention, inclusion and exclusion in daily life, opinion about the intervention, outcome of the intervention. Other codes were allocated in an inductive manner; themes that were not fully accounted for but proved relevant to the respondents were coded in an open fashion (Bryman, 2015). These codes can be seen as subcodes of the codes distilled from the literature and/or the theory-based description of the intervention. Loneliness, for example, proved to be an important topic for many respondents, and can be seen as a subcode of mental well-being. The data and the assigned codes were discussed among the three authors throughout the analysis. Consensus was reached through an iterative process of comparison and interpretation.

## Results

We found that the Communal Table was visited by four types of participants, which we referred to as “the lonely participant”, “the activist participant”, “the satisfied participant” and “the calculating participant”. These participants differed in the way they were or were not embedded in social networks and society. This also meant that they came in contact with the intervention in different ways and had different motivations to participate [Table 2].

**Table 2.** Type of participants and their social networks, motivations to participate and intervention outcomes.

	Network size	Motivation to participate	Found as a result of the intervention	Not found as a result of the intervention
Lonely participants (n=8)	Between 0 and 3 persons who visit regularly [mainly family: strong ties]	Finding lasting friendships [strong ties]	Temporary warm ties, temporary diversion from dense of loneliness	Lasting friendships [strong ties]
	4 respondents performed [unpaid] work [weak ties]	Overcoming loneliness and general lack of wellbeing		Overcoming loneliness and general lack of wellbeing
Activist participants (n=4)	Between 3 and 7 persons who visit regularly [family, partner or friend: strong ties]	Meeting other social groups and fighting the stigma of their own group through this [bridging capital]	Finding likeminded people to discuss views on care	Meeting other social groups and fighting the stigma of their own group through this [bridging capital]
	Active in client platforms/interest groups [strong and weak ties]	Finding a functional role co-determining the intervention [linking capital]		Finding a functional role co-determining the intervention [linking capital]
	All respondents performed [unpaid] work [weak ties]			
Satisfied participants (n=7)	Between 5 and 15 persons who visit regularly [family, partner or friend: strong ties]	Conviviality and good food	Conviviality and good food	-
	5 respondents performed [unpaid] work [weak ties]	Meeting new people and enjoying being around them [weak ties]	Meeting new people and enjoying being around them [weak ties]	-
Calculating participant	?	Low-priced food?	Low-priced food	?

### Reach of the intervention

The Communal Tables attracted a steadily growing number of participants during the study. This was seen as a success in itself by the organizers as well as the local authorities of the City of Amsterdam, which funded the project. There were four ways in which people came to participate in the intervention. (1) They had been informed of its existence by their social worker. (2) They had heard about it from a friend. (3) They had found out about it through flyers and/or posters. (4) They had contacted the organization about an activity or to get a buddy, and the organization followed this up by inviting them to join the Communal Table. Satisfied and activist participants often joined the intervention through their social workers and/or by being introduced by a friend. The lonely participants found out through flyers or were invited by the organization after there had been some activity-related contact.

### Experiences of participants

All the respondents of the communal table regarded the communal table as an enjoyable place. Respondents called the Communal Table “pleasant” “warm” and “welcoming” and respondents often said that “eating together is better than eating alone”. Most participants kept coming back during the 8 months of the field work. The variety in locations gave the Communal Table a “festive” character according to its participants. Participating meant that they saw new places, met new people and had an overall enjoyable time. However, the respondents’ experiences with the intervention were highly dependent on their own motivations to participate, which were in turn related to their personal situation and the degree to which they were embedded in social networks.

### The lonely participant

Lonely participants had a deficient network and expressed feelings of loneliness during the interviews. They were single, hardly had any family ties, and some of them had one or two friends, while some did not have anyone they would call a friend. They lacked strong ties; they did not have a social network of support and understanding. Some of the lonely participants had jobs, but this did not make them feel less lonely. Neither did having a job mean that they could befriend colleagues:

*Well that definitely doesn’t happen, hahaha* [on befriending colleagues]. *You come to work and then you go home*. (Andre, 63)

Lonely participants joined the Communal Table driven by their loneliness, looking for companionship:

*All I want is a real buddy. Someone I can get along with, do things with and talk about anything*. (Carolina, 51)

Like all participants, the lonely participants underlined the warmth of the atmosphere at the Communal Table. However, most of the lonely participants did not see or experience the intervention as an answer to their feelings of loneliness; it merely helped them to temporarily overcome these feelings. It functioned as a warm place in a cold society; a place where they were diverted from their daily problems and loneliness:

*I feel better there, but it’s just a momentary thing and then you’re gone, you know*. (Carolina, 51)

*It means I have something to do. Otherwise I don’t have anything to do anyway. Without it I would only sit at home and do nothing. I have to keep doing something. As long as I’m busy with something, then at least it takes my mind off things. (…) I feel a lot better when I’m there. Then I don’t ever want to go home again*. (Aysha, 23)

None of the lonely participants found a friend, in other words formed strong ties, at the Communal Table, whom they saw outside of the table. The respondents often mentioned the frequency and the time frame of the activities as reasons for this:

*No friendships are formed at all, I think. Because you come to eat and then you go home. That’s a shame. It is nice to meet each other like this but you don’t develop strong ties this way*. (…) *If it were every day, I would have made friends by now, haha. Yes, I think you should plan these activities a bit more often. Then it would go better and you’d get groups of friends, perhaps even couples*. (Andre, 63)

The group size and the lack of professional support aimed at forming friendships were also often mentioned as obstructing the formation of friendships:

*Well, it gets more and more busy. It makes me withdraw more and more. Smaller groups are fun and they allow you to make contact more easily. The bigger the group, the more it splits up into separate groups etc*. (Jacob, 53)

*Usually there are two social workers for a group of, say, twenty people. And if you’re only there for two hours from 5:30 to 7:30 pm, then two persons have to take care of twenty people. That doesn’t work*. (Jacob, 53)

The fact that friendships failed to materialize led to disappointment in some cases and various lonely participants stopped attending the Communal Tables:

*Often when I walk out the door* [of the Communal Table] *I’ve forgotten about it already. How it was. I completely forget about it immediately. I do try, but there will come a moment when I call it quits. (…) There are a lot of schemes to make people self-reliant, but they* [politicians and policy makers] *forget the people who need more help. And those people end up in the gutter, or up against the wall…this is how our society works*. (Jacob, 53)

The lonely participants spoke of a lack of mental well-being that was caused by their loneliness, in other words by their lack of strong ties. Participating in the intervention did not lead to enlarged strong-tie networks or increased societal participation of the participants outside the Communal Table. Hence, the lonely respondents did not report progress in terms of well-being or quality of life. They did find some warmth and temporary companionship, but they were unable to turn this into lasting strong ties.

Even though the Communal Table was not a complete solution to the problems experienced by the lonely participants, the warmth they perceived made them attend the Communal Table and continue to do so. Most of them did not have other similar regular societal activities they attended. Some respondents mentioned the higher prices of these activities as a reason for not visiting those:

*It’s cheap, one euro. I don’t really enjoy cooking for myself and you know, one euro for a meal is doable. And it is more fun to eat together than alone*. (Karima, 42)

All lonely participants mentioned the difficulties they had with attending new places and meeting new people in general. The perseverance of those organizing the Communal Tables in inviting possible and existing participants resulted in these people attending the intervention, even though it was a step that was considered frightening:

*I had something I guess, some contact with* [organization of Communal Table]*…a mail exchange or something like that. Anyway, something came up and because of this I kept receiving letters from them*. (Jacob, 53)

*I found it rather scary* [joining the Communal Table for the first time]. *But if I don’t make a move, I won’t get anywhere*. (Aysha, 23)

### The activist participant

The activist participants had more frequent contacts with family members or close friends, in other words strong ties, than the lonely participants. Some of them had a partner, but they were characterized mostly by being part of networks of weak ties. They were socially embedded in their neighbourhood and the wider society by participating in all kinds of initiatives and interest groups:

*We’re in a client interest group as well. We talk about changes and things that are not going well. Things we would like to improve basically*. (Sophie, 43)

In these interest groups or activities, the activist participants found a goal, a sense of meaning and a network of support. Often these groups and activities consisted of homogeneous groups with likeminded people who offered each other friendship and support, but at the same time these groups had an institutional character, with which it was attempted to bring about societal change:

*We’re developing a platform to spread our message, but also to answer questions from the municipal authorities, and discuss them together. So bottom-up. Not top-down*. (Jan, 46)

The activist participants were critical towards the Communal Table, as they were critical towards the contemporary care and social work discourse in general. They found that the stigma that attaches to people with an MID needs to be removed, so the activities should be mixed. According to them, people with a disability and “normal people” should eat together at the Communal Table, in other words they made a case for more bridging capital:

*Then they’ll find out that we’re normal people as well. And that there’s no actual difference. Perhaps we react a bit more slowly or talk a bit different but that’s it. (…) With the Communal Table you’re in a group. You’re attending a social event but you sit, with the group, at a separate table You’re not among the people and I find that…well not that I dislike it…but it’s a pity*. (Sophie, 43)

*That all groups come together. So normal people, in quotation marks, and us. But people with mental health problems as well. People with whatever background. I don’t care, as long as it’s mixed*. (Jan, 46)

The activist participants strove for a future in which everyone interacts with each other based on mutual respect. Through their activist attitude they built a network of kindred spirits, with weak to relatively strong ties, from social workers to clients, that helped them in their continuing struggle for acceptance. At the same time this social network functioned as a network of support for the activist participants. The Communal Table was a part of their active existence, a part that, just like the other aspects of social reality, can be improved and therefore needs to be criticized:

*If you start working from the top towards the bottom, if you draw up a plan on a drawing table and you impose it upon people (…) Well I’m sorry, but you have to turn this process around. You should ask the group first. I asked them* [the organization of the Communal Table] *that as well. We get the feeling that they’re going to talk about it with such and such a professional. Then I think, you guys are not living in the present*. (Jan, 46)

The activist participants were not visiting the Communal Table to enlarge their network and they did not need a nudge to increase their societal participation. Nor did they experience problems of mental health, and they were, for the most part, content with their lives. They did want to play a role in creating a more mixed and “better” Communal Table by turning it into a more “bottom-up” intervention. In other words, the activist participants aimed to become a functionally integral part of the organization, and their motivation to participate can therefore be described in terms of linking capital.

### The satisfied participant

The satisfied participants were embedded in a network of strong ties, mostly consisting of family. Most of them lived near family members whom they saw on a regular basis. Most of them had paid or unpaid work. The satisfied participants liked attending the communal table just as they liked going to other venues and activities. They often stated that the Communal Table was “very enjoyable” and “very pleasant”. They liked to have a good time and enjoyed interacting with others. The Communal Table was the perfect place for this and they tried to never miss one:

*It is great fun. You meet other people, from the neighbourhood as well. You’re in another environment for a change. You talk with each other*. (Hank, 42)

The satisfied participants were not lonely or attended the intervention to find new friends. They had friends, family and often a job. They visited the Communal Table because they liked social occasions, conviviality, big groups, new people and crowded places. Whereas the lonely participants were sometimes overwhelmed by big groups and the accompanying noise, the satisfied participants seemed to live by the principle of “the more the merrier”:

*Not that many people come to the Communal Table, right? It’s a small group actually. I think it would be more enjoyable with more people*. (Britney, 41)

There are many initiatives for people with MID, and the satisfied participants knew them all and frequently visited different community centres:

*I often eat at community centres. The ‘Robert Koch’* [community centre] *is near my house (…). I ate there yesterday and tomorrow I will eat there, and Thursday. I know a lot of people over there. That’s a lot of fun as well, yeah*. (Therese, 39)

The satisfied participants were content with their lives and did not feel lonely, excluded or in poor mental health. All the available activities, organizations and interventions together created a diverse and inclusive environment for them. The Communal Table can be seen as an enjoyable part of this social patchwork. The satisfied participants were interested in diversifying their network of weak ties, and they found opportunities to do this at the Communal Table. They enjoyed meeting and talking to people that did not necessarily need to become new additions to their existing strong tie network.

### The calculating participant

It was not completely clear if calculating participants actually existed, since none of the participants who were designated as such were willing to do an interview. What we found out about the calculating clients is thus based on observations, informal talks and the views of other participants about their existence. The participants perceived to be “calculating” seemed to visit the Communal Table mainly because of the low price of the food. They arrived just in time for the first course and left right after dessert.

The calculating participants often had partners. They hardly interacted with the other participants and when they did it often had to do with the “cheap food” that is “just one euro” or they asked if it would take much longer before the food was served. The calculating participants did not seem eager to find friends or become more active in their neighbourhood.

The calculating participant was a theme for the other participants at the Communal Table. They felt that there was a group that came solely for the low-priced food. It was especially the lonely participants who were not happy about the “existence” of these calculating participants. They wanted to form friendships, and the presence of a group that is not interested in this complicated their search:

*Yes for a euro they think, yes have a nice bite and adios. Hahaha, yes but I think that that’s what they’re coming for. I am sure actually. Yes, yes, yes they do talk with each other, but there’s the food and after that they’re gone*. (Andre, 63)

## Discussion

### Summary of findings

The Communal Table succeeded in reaching and attracting a diverse and loyal group of participants with MID. Based on their pre-existing social networks, and related motivations to participate, respondents could be differentiated into four groups. The satisfied participants saw the Communal Table as an enjoyable get-together; an event to create a more diverse network of weak ties (Granovetter, 1973). The activist participants criticized the homogenous [i.e., exclusively MID] character of the get-togethers, in other words the lack of bridging capital (Putnam, 1995). Activist participants also wanted to perform functional roles in the organization of the intervention that can be described as linking capital (Putnam, 1995). The calculating participants seemed to attend mostly because of the low-priced meals. The lonely participants, who had the smallest networks, felt that the intervention enabled them to temporarily not feel lonely, but that it did not help them to make new friends and form strong ties (Granovetter, 1973). Hence, although participants experienced conviviality and warmth, the Communal Table neither fulfilled its intended aims of enlarging social networks and/or increasing societal participation, nor did these intended effects match the motivations to participate of all participants.

### Strengths and limitations

A limitation of our sample is that the calculating participants could not be interviewed. We tried to include them in the study by offering a financial reward for participating in an interview at Communal Table C, but they had already left before there was an opportunity to invite people for interviews. In a way, this limitation derived from a strength of our study, since it was the participatory approach and the methodological triangulation that made it possible for us to find out about these “calculating” participants . If we had merely held interviews, we would probably have missed this type of participants entirely. The researcher’s “hanging around” (Bernard, 2011) helped to create a sense of trust and mutual understanding that led to rich data, and a deeper understanding of the complexity of the problems experienced by people with MID.

A second limitation is that we are not entirely sure about the representativeness of our sample. That is, irrespective of whether a reward was offered or not, half of the participants of all three Communal Tables declined our invitation for an interview. This means that—in theory—we may have missed another type of participant. However, the observations and informal talks of KK as participant-observer do not include any indication of such another group. Here again we think that our methodological approach has been of added value.

A final limitation of the study is that it is not entirely sure that all of the respondents had MID. Since we did not conduct IQ tests ourselves, it is possible that we included respondents with different kinds or degrees of disabilities. This could have led us to draw conclusions about people with MID based on data about people with a different kind or degree of disability. This being said, the intervention did specifically target people with MID, so it could be expected that the vast majority of the people participating in the Communal Tables and in the interviews could be described as having MID. Furthermore, most respondents were receiving help from the municipality directed at people with MID. Moreover, after being asked about this possible bias, the professionals of the Communal Table were rather positive about the homogeneity—regarding MID—of the group that participated in the Communal Table.

### Implications

Our findings indicate that interventions for people with MID that aim at strengthening social networks and societal participation should preferably be tailored to their pre-existing social networks and related personal motivations. This results in a more suitable supply of interventions to enhance the social network of people with MID. This means that lonely participants could have more personal attention and instrumental help with forming friendships, i.e., strong ties (Granovetter, 1973), and activist participants could be more closely involved in the organization of the intervention, i.e., linking capital (Putnam, 1995), and could perhaps even play a role in helping the lonely participants reach their goals.

The Communal Table appeared to be an inclusive approach, as a hard-to-reach group attended the intervention. This finding is supported by literature, which emphasizes the need for interventions to be located within the communities of people if they want to reach hard-to-reach groups (Fantuzzo, Stevenson, Kabir, & Perry, 2007), and that getting together for dinner is in itself a successful tactic to achieve this (Cortis, Katz, & Patulny, 2009). On the basis of our findings we can add the necessity to send invitation letters and to actively and repeatedly contact possible future participants. This is of crucial importance for people with MID, since they often have difficulties functioning in large groups and groups of new people, as we saw with the lonely participants.

We found that an intervention that aims at network enlargement and increasing societal participation simply by getting people to come together as a group is not a sufficient strategy. This is in line with the literature on group interventions for lonely elderly people [without MID] (Bodde, 1994; Jerrome, 1981, 1983). Jerrome (1981, 1983) concluded that lonely participants lacked the social skills to develop friendships. This makes us suggest that the inclusive character of the Communal Table could be combined with instrumental support programmes, such as social skills training and interventions to change activity patterns, since these programmes have been found to be most successful in terms of network enlargement for people with MID (Howarth et al., 2016). Such programmes could also address other skills that may be linked-up with the mental and physical health of people with MID, like learning how to select and prepare healthy food (Bennett & Cunningham, 2014; Johnson, Hobson, Garcia, & Matthews, 2011).

Alternatively, Karen Rook (Rook, 1984), contends that a group approach to counteracting social exclusion is actually a plausible one. However, she emphasizes that it is necessary to influence group dynamics and group interactions to support participants in dealing with the barriers that prevent them from social interaction and network enlargement. In the case of the Communal Table, our results indicate that stronger steering and supporting the group dynamics, facilitated by an appropriate group size and length of time spent together, may also contribute to the realization of network enlargement and increased societal participation of socially excluded people with MID. However, we also saw that which factors are experienced as facilitators may vary across groups of participants.

Finally, even though the ties at the Communal Table did not become as strong as is usually the case with family, friends and/or a partner, the warmth of such ties was simulated by the intervention. Perhaps it is more appropriate, in this light, to speak of “warm ties” than to speak of strong ties (Granovetter, 1973). The Communal Table to some extent created a nascent social network of support and familiarity, especially for those participants who lacked strong ties, allowing them to temporarily escape from their loneliness. Such a temporary relief may serve as a valuable starting point for participants in a strengthened version of the Communal Table to work on building the strong ties they may require to also improve their often poor mental health and quality of life (Berkman, 2009; Howarth et al., 2016; Rook, 1984).

### Future research

Our theory-based evaluation of the Communal Tables suggests that further research is needed on two topics. (1) Could a combined approach involving an inclusive group intervention and an instrumental personal intervention benefit the group with the smallest networks? (2) Which elements of interventions based on a group approach could be strengthened and/or altered to ensure that the interventions will benefit the participants more: what are the necessary ingredients for warm ties to turn into strong ties?

### Conclusion

This study shows that communal eating in the neighbourhood is an inclusive intervention that attracts a diverse group of participants. It is important that people with MID are not seen as a homogeneous group to create more effective social work interventions. Pre-existing networks play a role in the chances of success of interventions aimed at enlarging networks and increasing societal participation. Therefore, social network interventions for people with MID should be tailored to participants’ pre-existing networks and individual needs.
